# Digital barcodes of suspension array using laser induced breakdown spectroscopy

**DOI:** 10.1038/srep36511

**Published:** 2016-11-03

**Authors:** Qinghua He, Yixi Liu, Yonghong He, Liang Zhu, Yilong Zhang, Zhiyuan Shen

**Affiliations:** 1 Shenzhen Key Laboratory for Minimal Invasive Medical Technologies, Institute of optical imaging and sensing, Graduate School at Shenzhen, Tsinghua University, Shenzhen 518055, China; 2 Department of Physics, Tsinghua University, Beijing 100084, China

## Abstract

We show a coding method of suspension array based on the laser induced breakdown spectroscopy (LIBS), which promotes the barcodes from analog to digital. As the foundation of digital optical barcodes, nanocrystals encoded microspheres are prepared with self-assembly encapsulation method. We confirm that digital multiplexing of LIBS-based coding method becomes feasible since the microsphere can be coded with direct read-out data of wavelengths, and the method can avoid fluorescence signal crosstalk between barcodes and analyte tags, which lead to overall advantages in accuracy and stability to current fluorescent multicolor coding method. This demonstration increases the capability of multiplexed detection and accurate filtrating, expanding more extensive applications of suspension array in life science.

As an essential tool for diagnosis, gene expression and other life science fields, multiplex microarray has got rapid development in recent years^1^,^2^, especially for suspension array, which may be the most promising direction because of its performance in high-throughput and multiplexed detection^3^,^4^,^5^,^6^. For example, suspension array shows high sensitivity and achieves far lower detection limits in clinical diagnosis, such as detection of hormone and virus antibody, than traditional technology^7^,^8^. In genome and protein analysis, it also proves its capability in multiplexing^9^. The current suspension array was mostly encoded by luminescent barcodes, assembling organic fluorophores or luminescent quantum-dots with functional microspheres^10^. Technically, the functional microsphere can be identified by characteristic stimulated fluorescence spectra^11^,^12^. However, neither fluorophores nor quantum-dots can avoid the spectral overlaps in multiplexing, causing high constraints on decoding. Since the luminescent barcode is based on the waveforms and intensities of fluorescence peaks, both coding and decoding procedures require sophisticated operations, endowing barcodes distinctive characteristics of analogue quantity^13^. For example, the complicated deconvolving algorithm is required in decoding because of the unavoidable overlapping^14^. One more point, in current fluorescent multicolor method, both microsphere barcodes and analyte tags are based on fluorescent materials, causing high signal crosstalk in detection^15^. In consideration of above limitations, it is significant to build coding barcodes with more distinguishable coding signals.

In this manuscript, we report a kind of barcodes based on the LIBS technology, which may promote the arrival of the digital era of suspension array. LIBS researchers utilize the emission of plasma generated by laser pulse to provide nonintrusive, qualitative and quantitative measurements of elements in gas, liquid and solid samples^16^. Recent advances in LIBS have led to an extensive applications in different occasions, such as industry analysis, environment monitoring and medicine analysis^17^,^18^. Due to the linear characteristic of plasma emission, LIBS can provide wavelength data corresponding to elements directly, building the foundation of digital barcodes^19^. Meanwhile, the LIBS encoded polystyrene microsphere is designed with no fluorescence emission and the collection of LIBS spectra is independent from tag-fluorescence acquisition, eliminating the crosstalk between tag detection and barcodes decoding.

## Methods

### Optical method

**Figure 1:**
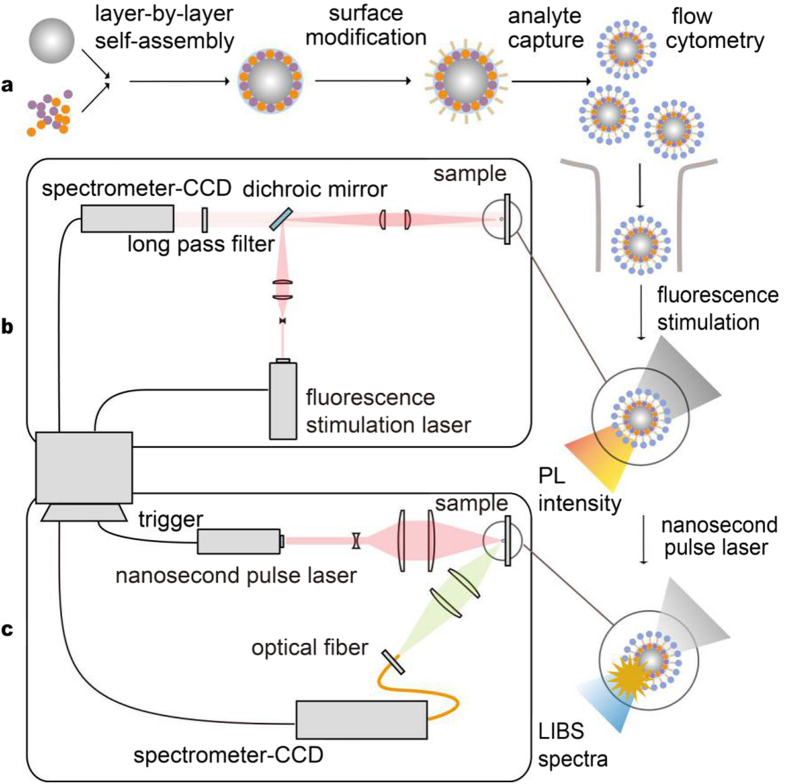
A schematic of LIBS-based coding system. (**a**) Simplified coding system of suspension array with added LIBS section. (**b**) Schematic of fluorescence intensity detection system. (**c**) Schematic of our home-built LIBS set-up.

Our coding system of suspension array was shown in Fig. 1a, the barcode is based on the LIBS encoded polystyrene microsphere (LIBS-PS), LIBS-PS can be utilized in sandwich immunoassays as capturing microspheres after surface modification^20^,^21^. Through a complete mixture and reaction with analyte, capturing microspheres enter into the flow system with fluorescence-labeled captive, such as proteins and genes. The microsphere experiences the laser stimulation of fluorescent tag on analyte and the detected fluorescence can prove the existence of captive. Then nanocrystals (NPs) on LIBS-PS were stimulated by pulse laser and the wavelength data of plasma emission can be collected to decode LIBS-PS, consequently accomplish the recognition of captive. A schematic of fluorescence detection system is shown in Fig. 1b.The laser beam (405 nm, 50 mw) was collimated by a 25.4 mm collimation lens and passed a 25 mm cylindrical convex lens. The beam was then reflected by a dichroic mirror (reflection band: 350–475 nm, transmission band: 492–950 nm) and focused by a 30 mm achromatic lens to form a line illumination on the sample. The fluorescence emitted from the sample was collected by the same achromatic doublet and transmitted through the dichroic mirror, then the light passes through the long pass filter (cut-on wavelength = 500 nm) to block the reflected laser. The spectrometer-CCD (SBIG, 3326 × 2504 pixels) system was put to detect the intensity of the fluorescence. The schematic of our home-built LIBS set-up is shown in Fig. 1c. Single pulse Q-switch Nd:YAG laser (1064 nm.180 mJ pulse-1, frequency = 1 Hz, pulse width = 8 ns), expended by a 20 mm focal length concave lens and then focused on sample surface by a 100 mm focal length quartz lens, generating micro plasmas on the LIBS-PS. Plasma light was collected through a fiber(62.5 μm in diameter, 1 m in length) after being focused by a 75 mm focal length quartz lens onto the entrance slit of spectrometer-CCD (Shenzhen Teksqray, 1024 × 1 pixels, resolution 0.35 nm). This set-up allowed recording the plasma emission in the 450–800 nm spectral range. The sample can form plasma on the surface immediately after being excited by pulse laser and the lifetime of plasma radiation mainly keep in 10 to 20 microseconds, thus we adopt external trigger mode to control the acquisition of plasma emission spectra, the integration time is set to 55 microseconds.

### Materials

**Figure 2:**
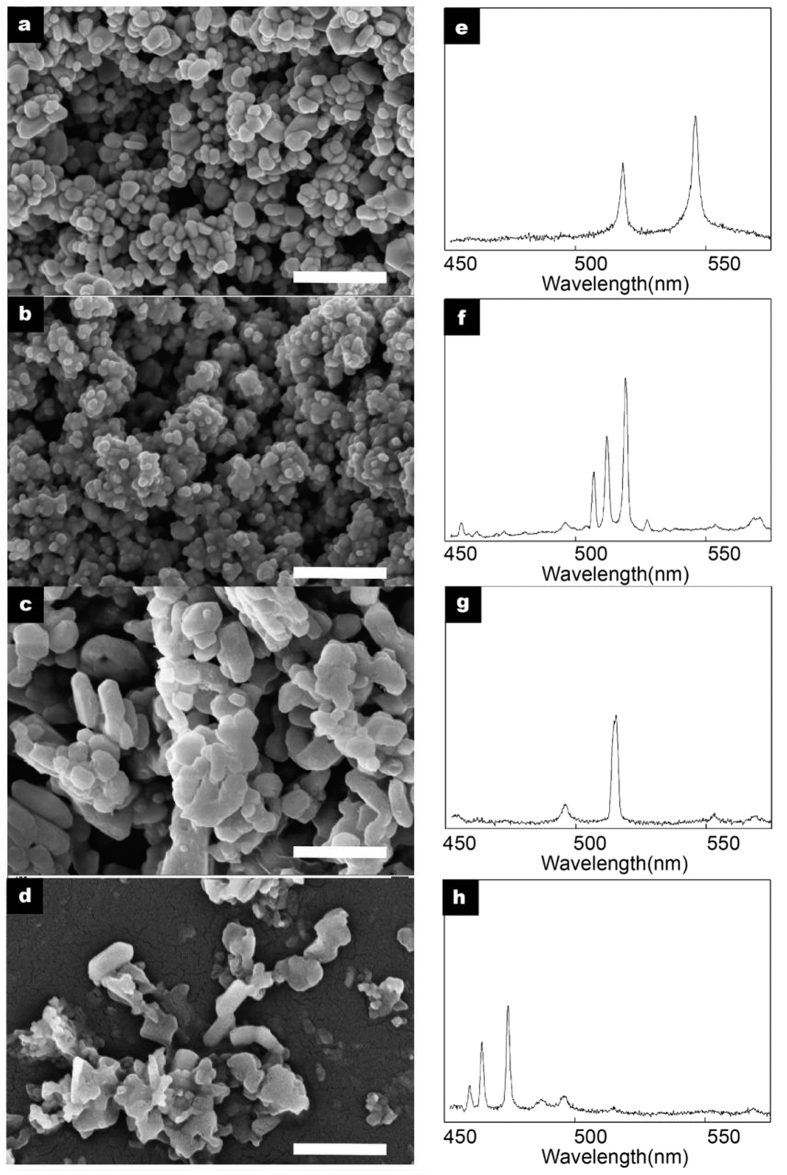
SEM images and LIBS spectra of NPs. SEM images of: (**a**) Ag NPs; (**b**) Cu_2_O NPs; (**c**) MgO NPs; (**d**) ZnO NPs. LIBS spectra of: (**e**) Ag NPs; (**f**) Cu_2_ONPs; (**g**) MgO NPs; (**h**) ZnO NPs. Scale bars are 200 nm for all SEM images.

Silver (Ag), cuprous oxide (Cu_2_O), magnesium oxide (MgO) and zinc oxide (ZnO) NPs (Macklin reagent, China, diameters = 40~100 nm) were used as characteristic LIBS coding materials in this letter (Fig. 2a–d). The plasma emission peaks we chosed as coding signals of three kinds of NPs are 520.9 and 546.5 nm for Ag; 510.6, 515.3 and 521.8 nm for Cu_2_O; 516.7 nm for MgO; 472.2 and 481.0 nm for ZnO. The plasma emission in the spectral range is stimulated and collected by our LIBS system, and wavelengths of emission peaks match well with standard data^22^ (Fig. 2e–h). We chose monodisperse polystyrene microspheres (Nano-Micro Research Center, Peking University, diameters = 10 μm) as LIBS coding carrier.

### Preparation of LIBS-PS

The layer-by-layer self-assembly method was carried out in the preparation of the LIBS-PS with some modifications^23^, showing as follow: 50 mg polystyrene microspheres (PS) were dispersed in polyethylenimine (PEI, Mw = 600000, 50 wt% in water) solution (25 mL, 4 mg/mL, 0.5 M NaCl), the mixture was allowed to react for 1 h with continuous stirring for the PEI adsorption on the matte surface of initial PS. The PEI@PSdispersion was then centrifuged and the supernatant was replaced with water. After redispersing PEI@PSby agitation and sonication for 5 s, the washing process was repeated at least two times to remove excess PEI. NPswere adsorbed onto PEI @PS by adding a sufficient suspension (0.2 g/L)of NPs in 2-propanol directly to thedispersion. After shaking the mixture vigorously for a few minutes, the non-adsorbed NPs were removed and the NPs-assembled PEI@PS (NPs@PEI@PS) was washed by three centrifugation/redispersion cycles (once in 2-propanol and twice in chloroform). The encapsulation of the NPs@PEI@PS was achieved by polyvinylpyrrolidone (PVP) and tetraethyl orthosilicate (TEOS). Sufficient PVP was dissolved by ultrasonication for 15 minutes in chloroform/2-propanol (9:1). The NPs@PEI@PSwere added to the solution and the mixture was sonicated for 10 seconds, then reacted overnight with continuous stirring. The beads were then centrifuged and redispersed in a solution of 4.2 wt% ammonia in 2-propanol. After this treatment, TEOS was added under continuous stirring and reacted for 12 hours to form final LIBS-PS. The total amount of PVP and TEOS depends on the desired thickness of the shell. PEI, PSS, PVP and TEOS were provided by Macklin reagent. Since the digital coding of LIBS-PS relies on the existence instead of the assembled ratio of different NPs, we chose the mixed solution of different NPs with simple ratio to provide coding materials. The types and ratios of coding NPs of 15 kinds of LIBS-PS are listed as follows: 1. Ag, 2. Cu_2_O, 3. MgO, 4. ZnO, 5. Ag/Cu_2_O(1:1, m/m), 6. Cu_2_O/MgO (1:1, m/m), 7. MgO/ZnO (1:1, m/m), 8. Ag/ZnO (1:1, m/m), 9. Ag/MgO (1:1, m/m), 10. Cu_2_O/ZnO (1:1, m/m/), 11. Ag/Cu_2_O/MgO (1:1:1, m/m/m/), 12. Ag/Cu_2_O/ZnO (1:1:1, m/m/m), 13. Ag/MgO/ZnO (1:1:1, m/m/m), 14. Cu_2_O/MgO/ZnO (1:1:1, m/m/m), 15. Ag/Cu_2_O/MgO/ZnO (1:1:1:1, m/m/m/m).

### DNA immobilization of LIBS-PS

As an efficient intermediary with abundant active catechol and amine groups, polydopamine (PDA) was coated on the surface of LIBS-PS (ZnO NPs assembled) to provide binding groups for DNA and other analyte^24^. The LIBS-PS were redispersed in Tris-HCl buffer (pH = 8.5), and the dopamine hydro-chloride (Alfa Aesar Co., Inc.) was dissolved in the solution with a concentration of 2 mg/mL. The mixture was stirred for 24 h at 37 °C. The PDA coated LIBS-PS were separated by filtration and centrifuged and washed with deionized water for several times. The target ssDNA (BGI, China) sequences were labeled with carboxyl groups modified water-soluble QDs (Wuhan Jiayuan Quantum Dot Technological Development Corporation, emission wavelength = 520 nm). The PDA coated LIBS-PS were dispersed in Tris-HCl buffer solution, followed by the addition of DNA-QDs. The mixture was stirred for 6 h at 57 °C. The solution was washed several times with 0.1 wt % SDS in phosphate buffer solution (prepared by NaH2PO4 and Na2HPO4, pH = 7.4).

### Multiplexed fluorescence imaging application of LIBS-PS in fluoroimmunoassay

To explore the performance of LIBS-PS in multiplexed detection, we carried out a two-color fluoroimmunoassay experiment.We selected LIBS-PS 1,4 to be capture microspheres after carboxylation. Mouse IgG (Beyotime Biotechnology)/Goat Anti-Mouse IgG (labeled with 525 nm QDs, Wuhan Jiayuan Quantum Dot Technological Development Corporation) and Rabbit IgG (Beyotime Biotechnology)/Goat Anti-Rabbit IgG (labeled with 605 nm QDs, Wuhan Jiayuan Quantum Dot Technological Development Corporation) are chosen to be antigen-antibody combinations.The experiment step are list as follow: First of all, 100 μL LIBS-PS 1 solution (10 mg/mL) was mixed with 1 mL phosphate buffer solution (PBS, 50 mM, pH7.4), 50 μL N-Ethyl-N’-(3-dimethylaminopropyl) carbodiimide hydrochloride (EDC, Aladdin Co., Inc.) solution (10 mg/mL) was added to the dispersion.The mixture was stirred for 15 minutes at room temperature. Then10 μL Mouse IgG solution (1 mg/mL) was added into the solution and incubated for 2 hours at 37 °C and the beads were washed with PBS by centrifugation for several times. 5% bovine serum albumin (BSA) solution (TBST, pH7.4) was used as blocking solution and mixed with beads for 1 hour to cover excess group. After washing for several times, the beads were redispersed in 1 mL PBS to form Mouse IgG-bonded LIBS-PS solution. The Rabbit IgG-bonded LIBS-PS solutionwas prepared with the same method. Finally, these two types of IgG-bonded LIBS-PS solution were mixed together, 10 μL Goat Anti-Mouse IgG solution(50 mM) and 10 μL Goat Anti-Rabbit IgG solution (50 mM) added into this dispersion. After incubation for 30 minutes at 37 °C, the beads were washed with PBS by centrifugation for several times.

## Results and Discussion

### Characterization of LIBS-PS

**Figure 3:**
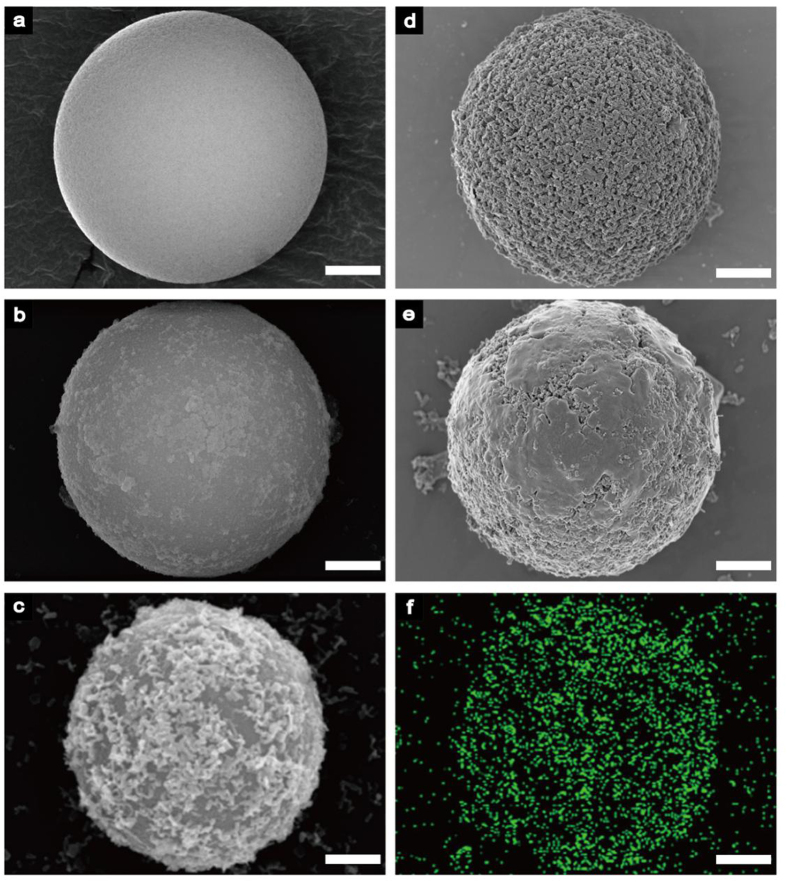
Experimental demonstration of the preparation of LIBS-PS. SEM image of **(a**) initial PS; **(b**) PEI@PS; **(c**) NPs@PEI@PS; (**d**) NPs@PEI@PS with PVPpassivation; (**e**) LIBS-PS with silica coating; **(f**) EDS image of the distribution of NPs (ZnO) assembled on LIBS-PS. Scale bars are 2 μm for all images.

Scanning electron microscopy (SEM, ZEISS SUPRA^®^55), energy dispersive spectrometer (EDS, ZEISS SUPRA^®^55), Fourier transform infra-red (FT-IR, Thermo Scientific Nicolet iS 50) spectra analysis, Zeta potential measurements (Zetasizer Nano ZS+MPT2)and transmission electronic microscopy (TEM, FEI TECNAI F30) had been used to characterize the resulted LIBS-PS. As shown in Fig. 3, SEM images described the surfaces of initial PS (Fig. 3a), PEI@PS (Fig. 3b), NPs@PEI@PS (Fig. 3c), NPs@PEI@PS with PVPpassivation (Fig. 3d) and finished LIBS-PS with SiO_2_ coating (Fig. 3e).The EDS image described the distribution of NPs (ZnO) assembled on LIBS-PS (Fig. 3f).

**Figure 4:**
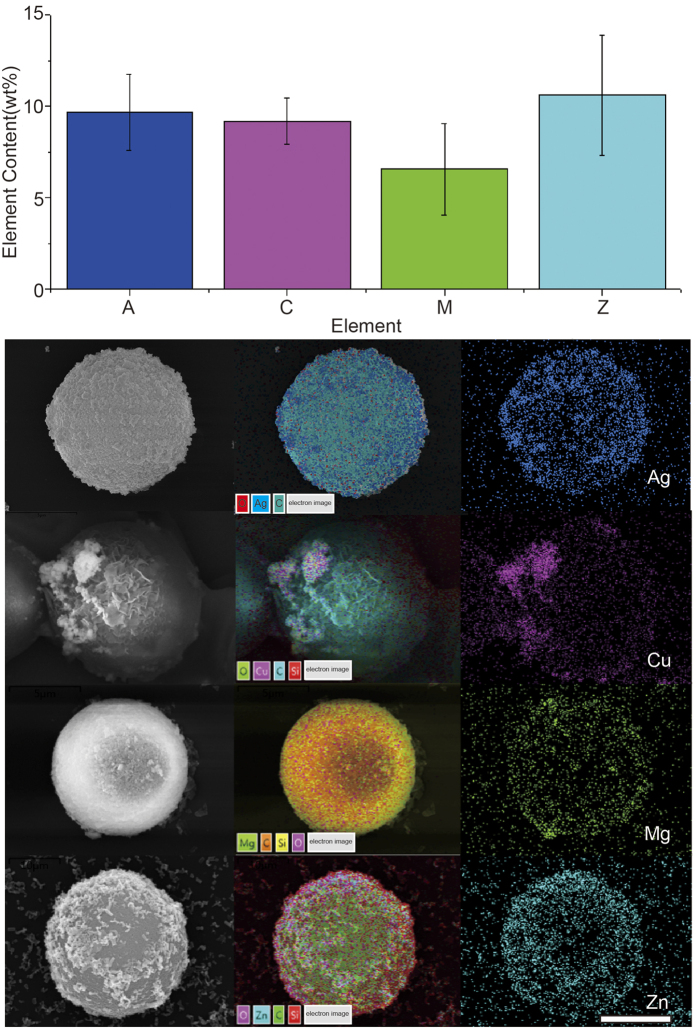
EDS measurements of various LIBS-PS. The upper histogram shows element contents, collected from a certain amount of beads, of different LIBS-PS, and the below image is the integration of SEM images, layered EDS images and EDS images of LIBS-PS 1–4. Scale bar is5 μm.

To demonstrate the reliability and reproducibility of self-assembling to LIBS-PS, the EDS measurements and infra-red spectra analysis were carried for LIBS-PS1(AgNPs@PEI@PS), 2 (Cu_2_ONPs@PEI@PS), 3 (MgONPs@PEI@PS), 4 (ZnONPs@PEI@PS). The histogram in upper table in Fig. 4 shows the data of mass content of coding element in different LIBS-PS, the below image shows the composition of SEM images, SEM-EDS coupled images and EDS images of four types of NPs@PEI@PS, it is obvious that the coding NPs were effectively assembled on various PEI@PS.

**Figure 5:**
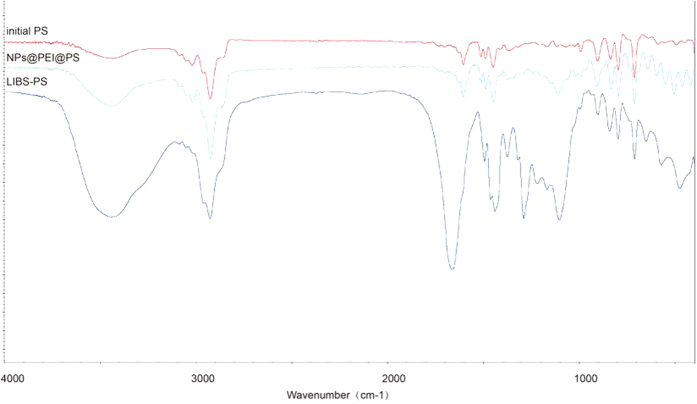
The fourier transform infra-red spectra analysis of LIBS-PS. The fourier transform infra-red spectra of initial PS (blue), NPs@PEI@PS (red) and LIBS-PS (purple).

The result of FT-IR spectra analysis is showed in Fig. 5, three lines represent initial PS, NPs@PEI@PS, LIBS-PS respectively. As we can see, the broad absorption peak around 3400 cm^−1^ in middle line proved the existence of PEI, the peak between 400 cm^−1^ and 600 cm^−1^ in middle line indicates that the NPs (ZnO) were assembled on PEI@PS, the absorption peak around 1650, 1500 and 1300 cm^−1^ in below line were caused by PVP coating, and the signal of SiO_2_ could be found around 1100 cm^−1^ in the below line. The results verified the existence of various chemical composition of coating.

**Figure 6:**
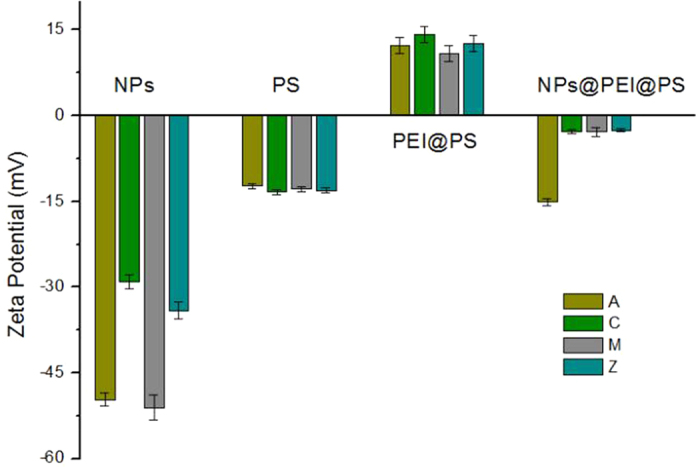
Zeta potential measurements in the preparation of LIBS-PS. Thehistogram showsZeta potential of initial materials (NPs and PS), intermediates (PEI@PS and NPs@PEI@PS) of LIBS-PS 1 (A), 2 (C), 3 (M), 4 (Z).

The Zeta potential measurements of each assembling states of LIBS-PS 1 (A), 2 (C), 3 (M), 4 (Z) were carried in Fig. 6, it is shown that the initial NPs and PS were both showing negative electricity. After decorating with PEI, four types of PEI@PS all turn to show positive electricity, which indicates that the PEI coating provides an appropriate electronic environment for self-assembling. The final Zeta potential of NPs@PEI@PS was negative, verifying that the NPs had been successfully assembled on the PEI@PS.

**Figure 7:**
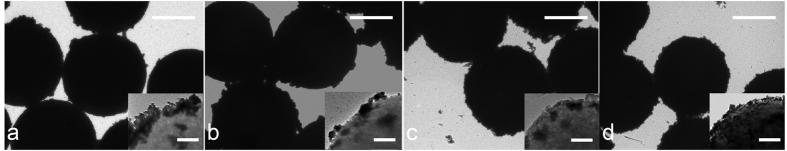
TEM images of NPs@PEI@PS. (**a**) Ag NPs; (**b**) Cu_2_O NPs; **(c**) MgO NPs 3; (**d**) ZnO NPs. Scale bars are 5 μm. The detail of surface are shown in the inset images and scale bars are 100 nm.

Finally, as shown in Fig. 7, TEM images showed the distribution of NPs on the surface of PS core for every type of sample. Obviously, different coding NPs can be assembled on the bead.

These measurements demonstrate the layer-by-layer self-assembly method is suitable to different types of NPs and is repeatable for LIBS-PS preparation.

### LIBS spectra and integrated digital barcode-map of LIBS-PS

**Figure 8:**
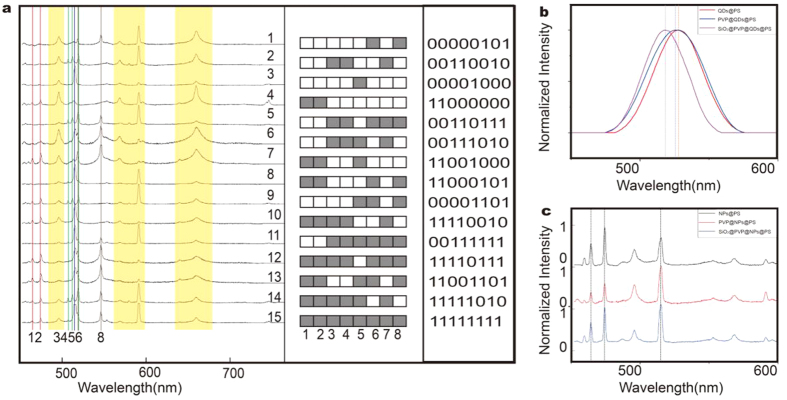
LIBS spectra and digital barcodes of LIBS-PS 1–15 and experimental demonstration of characteristics of the LIBS-PS. (**a**) LIBS spectra of 15 kinds of LIBS-PS, LIBS based digital barcode-map and operator sequences; (**b**) PL spectra of the QDs@PS, PVP@QDs@PS, and the SiO_2_@PVP@QDs@PS; (**c**) LIBS spectra of the NPs@PS, PVP@NPs@PS, and theSiO_2_@PVP@NPs@PS microsphere.

We made experiments of pulse laser stimulation on LIBS-PS 1–15, and plasma emissions were shown in Fig. 8a. In the measured spectral range, in addition to the common emission peaks of the basement in yellow frame, signal emission peaks match well with standard data of NPs, and they are sharp enough to read out peak wavelengths directly, substantiating the possibility of creating digital barcodes. Based on these eight coding emissions: 472.2, 481.0, 510.6, 515.3, 516.7, 520.9, 521.8 and 546.5 nm, we create a digital barcode-map and fifteen operator sequences for LIBS-PS. The colored plaid, corresponding to basic operator 1 in operator sequence, represents that coding emission is selected in the spectrum, and vice versa, blank plaid represents the unselected emission, corresponding to operator 0. According to this rule, the information of LIBS-PS were integrated into the digital barcode-map, creating fifteen operator sequences, as genuine digital barcodes to LIBS-PS. Also, LIBS-based digital barcodes can easily achieve multiplexing in high-throughput detection, to illustrate this viewpoint, a rough estimate is making as follows: Assuming that there are n kinds of element can be shown in the spectral range. We can obtain an approximate amount of barcodes with the operation of permutation and combination as follows:


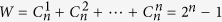


W represents the amount of barcodes. This is undoubtedly an enormous quantities, the practical amount may be much smaller while negative factors are concerned, such as spectral overlaps and emission deficiency. However, it is predictably that the amount of LIBS-based barcodes will appear an exponential growth with the increase of element types and expansion of spectral range, which is essential to the multiplexing performance of suspension array.

### Stability and potential of LIBS based coding

The peak positions of plasma emission hardly move with the influence of external factors, such as combined-state, chemical experience and physical state, providing LIBS another superiority in stability to luminescent multicolor coding, with a view of the fluorescence quenching and the blue/red-shift of peak positions caused by dimensional change and external disturbance^25^,^26^. To illustrate this point, quantum-dots doped microspheres (QDs@PS) (emission wavelength = 545 nm) were prepared with layer-by-layer self-assembly method and the fluorescence spectra were collected to make comparison with LIBS-PS. Obviously, both PVP and silica deposition lead to blue-shift of emission spectra (Fig. 8b), while the plasma peak positions of LIBS-PS can maintain stability in the total process of synthesis (Fig. 8c), which demonstrated the stability of LIBS-PS. Besides, the potential of LIBS-based digital multiplexing highly depends on the space of plasma emissions. The full width at half-maximum (FWHM) of emission peak is an important parameter to represent the potential of optical coding. Generally, smaller FWHM means less spectral overlaps and background interference, providing more coding space and accurate read-out data for digital barcodes. The FWHM of QDs@PS emission peak is between 40–50 nm while the LIBS-PS is less than 2–3 nm, shown in Fig. 8b,c. In other words, we narrowed down more than an order of magnitude of FWHM with the application of LIBS technology, providing enormous potential to LIBS based coding.

### Availability of LIBS-PS

**Figure 9:**
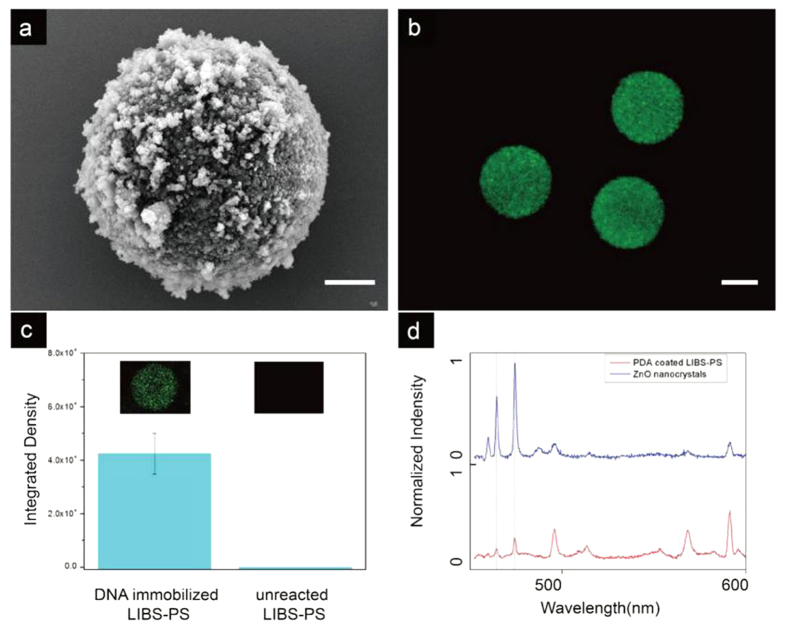
Experimental demonstration of DNA immobilization of LIBS-PS. (**a**) the SEM image of PDA coated LIBS-PS. **(b**) the confocal image of the DNA immobilized LIBS-PS. (**c**) the compare of PL intensity between DNA immobilized LIBS-PS (average = 42421.4, standard deviation = 8821.3) and unreacted LIBS-PS (average = 0, standard deviation = 0). (**d)** LIBS spectra of of the DNA immobilized LIBS-PS, the initial peak wavelengths of nanocrystal (ZnO) was put on the top of the image as standard. Scale bars are 2 μm for **(a)** and 5 μm for (**b**).

To verify the availability of the LIBS-PS in practical application, we made an experiment of DNA immobilization on LIBS-PS. We prepared PDA coated LIBS-PS as capturing microspheres (Fig. 9a), the QDs labeled target ssDNA sequences were utilized as analyte. After sufficient mixture and reaction, the DNA immobilized LIBS-PS was characterized by a laser confocal microscope (FV1000, Olympus), the image is shown in Fig. 9b. The average PL intensities of DNA immobilized LIBS-PS and unreacted LIBS-PS are shown in Fig. 9c, the existence of the target DNA can be proved by the detection of fluorescence intensity. The LIBS spectra of recorded LIBS-PS was collected by our system and the spectra is shown in Fig. 9d, the emission peaks match well with initial data. Hence, we achieved the whole detection link and demonstrated that the LIBS-PS is available in practical detection.

### Performance of LIBS-PS in multiplexed fluorescence imaging application

**Figure 10:**
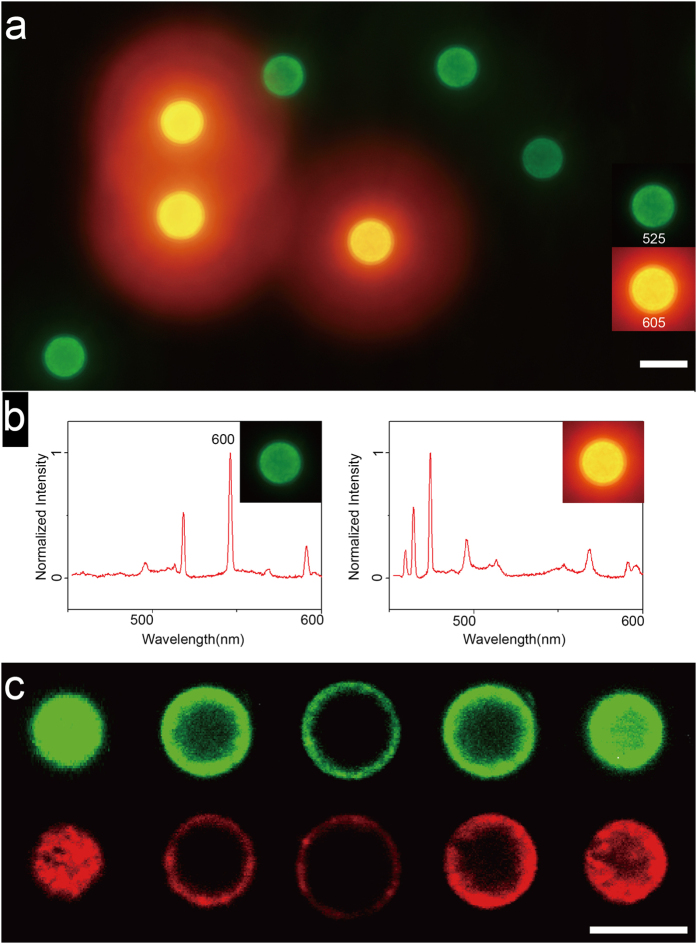
The performance of LIBS-PS in two-color fluoroimmunoassay. (**a**) the fluorescence microscopy image of two types of LIBS-PS which achieved specific detection of QDs labeled Anti-IgG. (**b**) the corresponding LIBS spectra of two types of LIBS-PS. (**c**) the laser confocal fluorescence sections of reacted LIBS-PS, the scanning increment was 1.75 μm. Scale bars are 10 μm.

Two-color fluoroimmunoassay experiment was carried to prove the performance of LIBS-PS in imaging application. As shown in the fluorescence microscopy image (Fig. 10a), there are two types of beads in the image, green beads were covered by QDs with 525 nm emission while the red ones were covered by QDs with 605 nm emission, which indicates that the QDs-labeled Anti-IgG had been captured by IgG-bonded LIBS-PS and every single LIBS-PS had been bonded with only one type of QDs. The corresponding LIBS spectrum was listed in Fig. 10b which has verified the specific bonding between barcodes and analytes. Consequently, these results demonstrate that LIBS-PS can successfully achieve specific detection to different Anti-IgG.

The crosstalk-resistant design of LIBS-PS is another essential innovation of suspension array. First of all, in the process of LIBS-PS based coding, we accomplished the collection of fluorescent tag on analyte first, and then collected the LIBS spectra to recognize the stimulated LIBS-PS respectively, which effectively separated the coding signal with analyte tags. Secondly, the coding material and chemical composition of LIBS-PS would not cause any fluorescence emission during the imaging application, which provided a clean background for fluorescence labeled analyte detection. We carried out laser confocal fluorescence section to reacted LIBS-PS in Fig. 10c, thescanning increment was 1.75 μm and sections shows that the fluorescence signal was pure and the inner LIBS-PS would not produce any fluorescence emission. Based on these strategies, we could accomplish tag detection without coding interference and digital coding without fluorescence background, which provides a more accurate detection and decoding in practical application.

## Conclusion

Based on above demonstrations, we confirmed that the LIBS-PS based digital barcodes of suspension array has several significant advantages. (1) Plasma peaks of the LIBS-PS are sharp enough to achieve digital coding with read-out wavelength data, highly promoting the coding accuracy of suspension array. (2) The amount of barcodes can obtain an exponential growth with the increase of element types and expansion of spectral range. (3) The location of plasma peaks can keep stable on various occasions, promoting the accuracy and stability of barcodes. (4) Benefited from special design of LIBS-PS and the respective signal collection of barcodes and analyte tags, we can obtain clean signal of tag detection and decoding with this method. (5) Since the FWHM of LIBS-PS emission peak is more than one order of magnitude lower than current coding spectral, the spectral space is released for a large amount of barcodes, consequently promoting the performance of multiplexing. In fact, we can totally achieve much smaller FWHM than 0.5 nm if peak-broaden affecting elements, such as self-absorption, matrix effect and the initial continuous spectra, are eliminate^27^, then the performance of digital multiplexing can get an obvious promotion.

In summary, we have demonstrated a successful digital multiplexing method of suspension array based on laser induced breakdown spectroscopy technology. We introduced the preparation and performance of LIBS-PS as the foundation of digital multiplexing. The compare with luminescent barcodes-based coding method illustrated that the LIBS-PS based coding method can create digital barcodes without fluorescence background, and own overwhelming advantages in accuracy, stability and multiplexing performance. Although there are still some limitations in practical application, for example, LIBS stimulation would destroy the whole sample, which hinders additional experiments or manipulation, it is still reasonable to consider that the LIBS-PS based digital barcodes can be a leap-style development of suspension array and play more important role in multiplexed detection over the coming decades.

## Additional Information

**How to cite this article**: He, Q. *et al*. Digital barcodes of suspension array using laser induced breakdown spectroscopy. *Sci. Rep*. **6**, 36511; doi: 10.1038/srep36511 (2016).

**Publisher’s note:** Springer Nature remains neutral with regard to jurisdictional claims in published maps and institutional affiliations.
